# Sustainable care coordination: a qualitative study of primary care provider, administrator, and insurer perspectives

**DOI:** 10.1186/s12913-019-3916-5

**Published:** 2019-02-01

**Authors:** Mark D. Williams, Gladys B. Asiedu, Dawn Finnie, Claire Neely, Jason Egginton, Lila J. Finney Rutten, Robert M. Jacobson

**Affiliations:** 1 Mayo Clinic, 200 1st St SW, Rochester, MN 55905 USA; 2 Institute for Clinical Systems Improvement, Minneapolis, MN USA

**Keywords:** Case management, Delivery of health care, Health services research, Insurance, Qualitative research

## Abstract

**Background:**

Care coordination has been a common tool for practices seeking to manage complex patients, yet there remains confusion about the most effective and sustainable model. Research exists on opinions of providers of care coordination but there is limited information on perspectives of those in the insurance industry about key elements. We sought to gather opinions from primary care providers and administrators in Minnesota who were involved in a CMS (Center for Medicare and Medicaid Services) transformational grant implementing COMPASS (Care Of Mental, Physical And Substance-use Syndromes), an evidence-based model of care coordination for depressed patients comorbid with diabetes and/or cardiovascular disease. We then sought to compare these views with those of private insurance representatives in Minnesota.

**Methods:**

We used qualitative methods to conducted forty-two key informant interviews with primary care providers (*n* = 15); administrators (*n* = 15); and insurers (*n* = 12). We analyzed the recorded and transcribed data, once de-identified, using a frameworks analysis approach.

**Results:**

We identified six primary themes: 1) a defined scope, rationale, and key partnerships for building comprehensive care coordination programs, 2) effective information exchange, 3) a trained and available workforce, 4) the need for a business model and a financially justifiable program, 5) a need for evaluation and ongoing improvement of care coordination, and 6) the importance of patient and family engagement. Overall consensus across stakeholder groups was high including a call for payment reform to support a valued service. Despite their role in paying for care, insurance representatives did not stress reduced utilization as more important than other outcomes.

**Conclusions:**

Primary care providers and administrators from different organizations and backgrounds, all with experience in COMPASS, in large part agreed with insurance representatives on the main elements of a sustainable model and the need for health reform to sustain this service.

**Electronic supplementary material:**

The online version of this article (10.1186/s12913-019-3916-5) contains supplementary material, which is available to authorized users.

## Background

The healthcare system in the United States lacks universal insurance coverage for citizens such that patients pay for health goods and services via a mixture of employee-paid insurance, government-based insurance (for specific populations), privately purchased insurance, or out-of-pocket payments. Government-based insurance is largely divided into Medicare (largely an age-based federal service program guaranteeing coverage for those 65 and older) and Medicaid (a public assistance program varying by states for needy Americans of all ages). Healthcare providers have largely been paid on a fee-for-service model which is felt to be part of the explanation for rising costs. Health care delivery systems face a difficult time in the quest to innovate for improved value (defined as better outcomes for less cost) in that there is no payment for these new models in a fee-for-service system. Thus, healthcare systems pulling their resources into value-based delivery must bear the associated cost while any savings achieved in reduced utilization benefit insurance companies.

Care coordination is a model of healthcare delivery created out of the growing awareness of the prevalence and cost of chronic conditions among patient populations [1], and the fragmentation [2] and limitations of a healthcare system organized around fee-for-service and acute care [3, 4]. Encouraging evidence has accumulated that care coordination frequently leads to improved clinical results and in narrow circumstances, improved cost outcomes [5–7]. However, in the complex world of routine clinical practice there are at least 40 definitions of care coordination [8] resulting in confusion as to what components of care coordination are necessary to attain desired outcomes. For nearly a decade there has been recognition that a major barrier to sustaining care coordination is a lack of a viable financial model of support [4, 9]. Medicare began offering care coordination reimbursement in 2015 for aspects of care coordination, however system barriers led to a less than 5% uptake of these billing codes within the first 15 months [10]. Reorganizing care delivery without a reimbursement or cost sharing model to cover care coordination is a significant expense, and there are noteworthy examples of programs investing time and money on care coordination without obvious return on investment [11]. For care coordination to succeed in a fee-for-service world where patients depend on commercial insurance, insurers must recognize at least some levels of care coordination as a reimbursable service [12].

In 2012, the Institute for Clinical Systems Improvement (ICSI) was a recipient of a 3-year Centers for Medicare and Medicaid Services (CMS) innovation award entitled Care of Mental, Physical and Substance-use Syndromes (COMPASS) aimed at implementing an evidence-based model of care coordination for depressed patients with diabetes mellitus and/or cardiovascular disease. The CMS innovation grants were part of the government’s efforts to invest in healthcare changes that might demonstrate ways to move from volume based models to value based models while funded by the research grants. The model was adapted from Wayne Katon’s TEAMcare model originally described in 2010 [13]. In brief, this model involves dedicating trained care coordinators to enroll and follow complex patients with the above conditions with weekly review of these patients by a team that includes a psychiatrist and a primary care physician. The patients are monitored on outcomes related to these chronic illnesses--blood pressure, glycosylated hemoglobin, and the PHQ-9 (Physician’s health questionnaire, 9-item) in a clinical data registry and recommendations are routinely made to the patient’s primary care team in order to adjust treatment to target outcomes of establishing control of these chronic conditions thus allowing return of these patients to usual care. To be eligible for the program, adult patients needed to have a diagnosis of depression (any of the DSM-5 diagnoses) with a PHQ-9 score of at least 10 and evidence of poor control of diabetes and/or cardiovascular disease. ICSI translated this model into 174 clinics in 18 medical organizations in 8 US states including Minnesota [14]. The sites were encouraged to keep as close to the model as possible, but as this was a quality improvement oriented translational effort versus an outcome-based project, sites were also able to adapt the model to local needs. The effort was successful in improving clinical outcomes [15] but with no viable insurance coverage for care coordination at the time of this study, each participating organization was faced with what to do with the progress they had made during the grant once funding ceased.

Care coordination and attending to behavioral health needs of populations of patients with chronic health conditions are both key components of the majority of health reform models in the US. COMPASS was a rare example of a care coordination model that simultaneously addressed a mental health condition and chronic medical conditions with good outcomes in real world settings. To sustain the model past the grant funding, practices needed to justify the costs. The purpose of this study was to capture the perspectives of primary care providers and administrators having recently implemented a successful evidence-based model of care coordination (COMPASS) in Minnesota and to contrast these perspectives with those of insurers in Minnesota most familiar with models of care coordination. Our expectation was that providers would favor improved clinical outcomes over cost reduction and insurance company representatives would have the reverse opinion. To our knowledge, this is the first such study to include and contrast the input of insurers with those inside clinical practices.

## Methods

### Study design and setting

This study used an applied qualitative health research approach [16] focusing on stakeholder’s views on care coordination for complex patients, in regards to optimal inclusion and exclusion criteria, optimal components of care coordination, critical outcomes for such programs, facilitators and barriers and how that can be applied to sustain care coordination programs in health care setting. The Institutional Review Board for Research on Human Subjects at Mayo Clinic approved the study protocol and all respondents provided informed consent. All relevant aspects of the methods have been evaluated using qualitative checklist, COREQ32 (Additional file 1) [17].

### Respondents and recruitment

We identified primary care providers via study data, tracking the number of patients referred per provider into COMPASS to identify providers with at least 4 referrals into the program. Efforts were made to mirror the percentages of interviewees from medical groups with the percentage of patients in COMPASS from that group. Administrators were identified from COMPASS sites by one of three possible roles: (a) those responsible for hiring and/or managing care coordinators; (b) those responsible for overseeing a clinical budget; or (c) those responsible for the overall direction of population health management in a health system. We identified insurers beginning with information on market share of the private insurance market obtained from the Minnesota Department of Health Economics Program analysis of the Minnesota Comprehensive Health Association (MCHA) premium database for 2013. We reached out to insurance groups with the largest market share first for contacts. Insurers were identified through three sources: (a) ICSI insurance groups board members; (b) Mayo Clinic contracted insurance providers; and (c) participating insurers’ own identification of additional potential participants.

We used a purposive sampling approach using criterion-based and snowballing techniques to recruit respondents for the study. By this approach we initially approached stakeholders with firsthand COMPASS experience via email, who then provided references for other potential respondents, who then recommended additional respondents and so on. This process ensured that stakeholders with no knowledge or experience with care coordination or COMPASS were eliminated, and only those who were best suited to meet the purpose of the study were included. We sent recruitment emails to the identified stakeholders explaining the purpose of the study, that is, to understand their unique perspectives on critical aspects of a sustainable model of care coordination for complex patients. Also the recruitment email indicated that if they choose to participate their contact information will be given to SNG Research Corporation^1^ to schedule interviews. We asked stakeholders to respond to the email or call the study coordinator if they wish to participate.

We identified a total of 84 primary care providers and invited them, of whom fifteen agreed to an interview. We identified a total of 26 administrators, 15 of whom participated. All 12 insurers that we identified agreed to an interview. We submitted the list of respondents and their contact information to SNG Research Corporation who then scheduled and conducted the interviews. We obtained informed consent from all respondents orally.

### Data collection

Using a standardized semi structured interview guide developed by the research team, SNG research group conducted individual interviews with identified stakeholders over a period of three weeks (April–May 2016). SNG approached each stakeholder group with the same set of questions regarding key components of a sustainable model of care coordination in a setting of limited resources. We have attached the interview guide for reference (Additional file 2). ASNG conducted all interviews by phone and transcribed the audio recordings. SNG transferred all audios and de-identified transcripts via a secured drive to the Mayo Clinic Qualitative Research Unit whose personnel include authors GBA, DF, JSE. We monitored data saturation continuously throughout the interview process. For comprehensiveness we chose to fully interview all respondents who agreed to participate to reduce the chance of missing any ideas.

### Analysis

Investigators included experts in population health sciences, health care policy and research, sociology, health services research and qualitative research methods. SNG conducted interviews and transcriptions but they did not analyze the data. We proofed all transcripts received against audio files by QRU for consistency and accuracy. Four QRU analysts (GBA, DF, JSE then coded, analyzed, and interpreted the transcripts using a framework analysis approach [16–18], supported by qualitative analysis software (NVivo 10.1, QSR International Pty Ltd.). They analyzed the data using an integrated approach of inductive codes emerging from the data and a priori codes (derived from interview questions) [19]. This process involves being “immersed” in the data, identifying preliminary themes, and developing a coding framework, then applying this framework back to the verbatim transcripts. Preliminary analysis included multiple initial readings in order to obtain a sense of the whole data. DF, GBA and JSE first independently read the few transcripts, and performed initial coding of the data. This meant that they independently ascertained stakeholder opinions regarding the common understanding and components necessary for sustaining care coordination paying attention to emerging themes and then guided by the interview questions. This led to the identification of preliminary themes within the data and similarities were noted across transcripts. This analysis approach is an interpretive process whereby patterns are identified by systematically reviewing the data to elicit common themes, without generating theory [18]. Framework analysis is particularly suited to projects like these, as we sought to analyze cross-sectional data among stakeholder groups. Inter-coder agreement allowed for discussions on discrepancies in the coding until consensus was achieved in the final results.

## Results

Stakeholders interviewed for this study included administrators (*n* = 15), insurers (*n* = 12), and providers (*n* = 15). The interview length averaged 22 min. We report themes that emerged as important among stakeholders, highlighting common understanding and components necessary for sustaining care coordination. Data analysis yielded six main themes and categories:

- *A defined scope, rationale, and key partnerships for building comprehensive care coordination programs*
- *Effective information exchange*
- *A trained and available workforce*
- *The need for a business model and financially justifiable program*
- *Evaluation and improvement of care coordination programs*
- *Patient and family engagement*

For each category we present the barriers that were reported by stakeholders as well as facilitators and components to a successful care coordination program. Example quotes from stakeholder groups are illustrated in Table 1.

**Table 1:** Themes and Representative Quotes for Sustainable Model of Care Coordination for Complex Patients

Themes	Stakeholder Quotes
1.A defined scope, rationale, and key partnerships for building comprehensive programs *1.1 Care coordination Inclusion and exclusion criteria*	A1: *“There’s kind of a tension between whether we should be caring for the sickest of the sick …as we are thinking about with chronic disease where whether there is more of a place to be caring for what might be considered a rising risk population… There needs to be defined criteria and there needs to be a defined end point because…it would* [be] *nice for patients to be in the program forever…[but] you might be stuck in the cycle of not progressing. Then the rest of your patients might suffer from that by not being able to get into the program.”* I1: *“We have to make sure we can segment our population and understand how to identify the right people for case management or care coordination... The appropriateness of identifying people for care coordination.*”
*1.2. Care coordination as a vehicle to bring together medical and community resources for complex patients*	P2: *“Ideally things get straightened away and people can graduate but these patients are complex for a reason and they might have relapses for different things so I think always having a pretty accurate assessment is a good idea for a lot of them anyway.”* A10: *“The thing that we also struggle with is the complexity of the psychosocial conditions that the patients present with…I would envision that it would be a multidisciplinary team...If it’s more psychosocial in nature, then a social worker is probably more appropriate. It would be a model in which there were multiple participants. So the care coordinator, there would be mental health support, there would be medical support, there would be social work involved, pharmacy. That [multi-disciplinary] team, while there are distinct roles and responsibilities, would also know how to work collaboratively with each other in the areas where things might overlap. An example, there’s things that are different about the RN and social work role, but there’s places where it [their roles] might overlap.”* I1: *“There has to be a comprehensiveness of buy in. If you are creating a care team approach within a care delivery system, everybody has to buy in to… we’re doing team-based care. If you don’t have that type of accountability, and you don’t have folks that truly believe in team-based care, it’s not going to work. It’s going to fail…Making sure everyone is on the team so to speak… that’s the biggest critical success factor.”* *P14: “Ideally have an effective team that can address all of the patient’s chronic health issues without diluting the expertise needed to manage it.”*
2. Effective Information Exchange *2.1. Strong Interpersonal level-interactions among stake holders- and with patients*	P11: *“The hospital may have a nurse navigator that is helping take care of the patient in the hospital, but the communication back to us isn’t very good. The nursing home may have a social worker but the stuff they are doing there communicating back to us doesn’t happen. There are holes in our system that as a provider, as a team, if you said had I only known that, I would have done something different.”* A5: *“I think co-location of the care coordinating group or team and the medical home team. In a perfect world, you would want them co-located because that facilitates communication. In smaller clinics or in rural areas, that’s not going to be feasible so we would somehow need to leverage technology to make sure that there is enough contact between those groups.”* I3: *“Good communication between the medical staff and the case manager, and good information flow between the specialists and the case manager.”* I12: *“With all medical care, there has to be a longitudinal, trusting relationship that occurs between the care team and the patient.”*
*2.2. The need for data and access to an interoperable health information system*	I4: “*There needs to be very intense efforts…to engage the client in care coordination... It’s [care coordination] required that the appropriate amount of trust and effort is built in order to get the client to engage.”* A4: *“There needs to be a place to keep this [care management] information so that people can readily access it when the patient makes contact with the care team.”* A10: *“There needs to be a plan of care and that plan of care needs to be accessible not only by the primary care team but every other member of the team whether it’s within the same health care system… the inpatient units need to be accessing it [plan of care], everybody needs to know how to use it. People need to be able to contribute to it [the plan of care]to help everybody be on the same page. In the perfect world we’d be able to also do that same thing with any other community entity user, external agencies that the patient is working with as well so we are all working off that same platform.”* P9: *“If we can set up web access so you can do face-to-face coordination but through the internet, if they are available for phone systems or something like that, like Skype. I think sometimes it’s too easy for folks to just spend a few minutes on the phone and if we can actually see a patient face-to-face, even if it’s over a camera…that’s just an extra step…using communication that’s available and technology as well to help us.”* I7: “*From a technology standpoint, one of the biggest challenges is getting systems to talk to each other. Having an electronic medical record be able to communicate with a claims system. That’s the concept of big data… Making sure that the health system and the health plan and the community resources are connected and talking to each other on behalf of the patient so that information that is shared, recommendations that are given, are consistent and supportive of each other so at the end of the day the patient is well served.”*
3. Trained and Available Workforce	I11: *“If it’s [the care coordinator is] a nurse, she has got to be very skilled and knowledgeable and passionate about not only the medical side but the patient interaction and advocating and understanding what the social connection resources are required to get that patient through.”* *P8: “Getting well-trained individuals. Getting individuals who stick with it [care coordination] for years and so as they further develop their expertise as a care manager as opposed to finding an easier, more fulfilling job somewhere else.”* P1: *“Having somebody that has the understanding of those [disease] conditions…is going to be much more beneficial than perhaps other clinical conditions. There has to be an understanding of what are the major morbidities and comorbidities in the area and have peer coordinators that have a good background on those conditions… You need to have a reasonable ratio of patients to care coordinators so the care coordinator herself or himself doesn’t feel burned out and it becomes the weakest link in the process.”* P6: “*You need to show some stability in the personnel that provides care management. Nothing puts off patients or referring physicians if you see a carousel of people involved with your patients. There needs to be stability in the program and the personnel providing the program.”* I12: *“So somebody that is able to interact with patients wherever they’re at or at whatever level they need to understand basic concepts, like motivational interviewing, to effect behavior change if that’s the issue and also have a grasp of what other adjunct services are available so if that means engaging county social workers or whatever other programs are out there, that they understand at least how to work through people that are out there that know how to do that sort of thing.”* A12: *“If you start to introduce lower levels of education and individuals into care coordination, then you need to have a clear scope of practice so that they are not tip toeing into areas that may not be appropriate… Individuals who are not licensed or not an RN really should not be making clinical assessments or giving clinical advice because that’s way outside their scope. Nor should they be doing any kind of therapy or counseling. But what we see is that individuals who are not RNs don’t know that… Clear boundaries, clear parameters of what is okay and what is not okay.”*
4. Need for a business model and financially justifiable program	A5: *We need to make a substantive difference in utilization of health care. If we can’t do that, we can’t pay for this[care coordination] and if we can’t pay for this, it’s [care coordination is] not going to exist. Nobody wants to talk about that [who pays for care coordination services]. That’s like taboo to talk about that but at the end of the day, this thing [care coordination] isn’t going to fly if it doesn’t make economic sense.* I11: *“They try to tack it [care coordination] onto something else, they under-source it, they don’t have sustainable funding for it as a pilot, and there are a lot of other competing interests especially in a primary care provider’s office where they’ve got 150 things that are the most important thing to measure and manage and that’s never going to work.”* A11: *“I think there has to be an aligned financial model and an aligned practice model for that [care coordination] to work well... This [There] was a grant-funded study that covered part of the cost to introduce these care coordinators into our system but in a fee-for-service kind of reimbursement mode there’s not a lot of incentives or a strong business case for doing some of this work… If you have an aligned financial model where the institution, the provider and the patient all have aligned incentives to do these [care management] things, that’s key.*” A1: *“I say 50% of our enrolled patients drop out because of that [finances]. They have to stop cost sharing. It [care coordination] is either a part of a wellness benefit to improve wellness or it’s not. If it’s not, don’t expect people to be care managed because they will opt out. They[policy makers] have to stop cost sharing*.” P4: *“Someone has to pay for it [care coordination services]. The insurers have to pay for it. We can’t do it if we do not have the resources; primary care is not going to be able to do it all. It has to be paid for.”* P13: *“Obviously cost on the side of the patient because they[patients] are being recommended to do things that involve more medications or visits.”* P13: *“Also on the side of the health care organization, the cost of the personnel and time it takes [coordinate care].”*
5. Evaluation and Improvement of Care Coordination Programs	A10: *“We would have a way to have solid metrics too that could help measure success that would be focused around the triple aim. The satisfaction of the patients, providers, nursing staff, everybody that’s working as a part of that support team, and then of course cost and clinical outcome data as well.”* A2: *“We need some better data. That’s what a lot of us hope and COMPASS overall will be able to provide data that if you build it, they [patients] will come. If you do this well, you will either attract more patients, which will help sustain your organization. Or the data will prove that, indeed, investing in care management services has an outcome that we can measure. I don’t think that’s there yet.”* I7: *“Having an electronic medical record be able to communicate with a claims system. That’s the concept of big data. Bringing claims information and EMR data together to be able to evaluate, to analyze, to stratify and really use that to kind of try and do prescriptive care coordination that’s specific to a particular patient and really prospective as opposed to retrospective.”* I12: *“From a patient perspective I think you want to ask those quality of life questions: ‘am I able to do the things I want to do, am I happy, do I feel like I’m at the best health that I could possibly be.’ So patient reported outcomes. And then there are utilization outcomes so total cost of care, and those other preventable episodes of care…. And then of course the big dots, life expectancy, mortality rates...”* P3: *“Research certainly is an important part. To be able to successfully implement it [care coordination program] you have to periodically be able to evaluate the process, evaluate outcomes, make changes. Those pretty much in terms of how it [the program] can be successful and of course be able to structure or formulate an effective model. The model itself is a major factor to decide whether this care coordination will be effective or not.*” P5: “*A rigorous method to assess need, to intervene to make choices what may be appropriate, to intervene or work with the patient on and then assessing effectiveness and then returning to taking that assessment back in and deciding whether we proceed forward or not…I sometimes feel like we talk about a lot of things but we haven’t found an effective way to move patients forward.”*
6. Patient and family engagement	P13: *“If patients are in a mental health crisis and can’t access that care, they are unlikely to be managing their chronic disease, physical disease as well, or to be in contact with a care coordinator.”* P14: *“Some hesitancy based on patient’s perspective being at this point that’s a non-traditional approach to chronic care so they [patient] may not be keen to do it[care coordination] so that may be an issue.”* A9: “*Patients have to be willing participants as well…we certainly found patients that are very eligible but really don’t want to participate…is patients who really need help but are not interested. How to engage patients is a barrier…. Patients to be willing to participate in their care as well by making themselves available for care coordination.”* I9: *“First they [patients] have to be agreeable to engage in it [care coordination program]. If they are not going to be compliant or they’re not interested, we wouldn’t engage them in case management. Sometimes the member may refuse, and the family members are participating. So we work with family members. Somebody on the other side has to be participating.*” A10: *“I think part of the problem we sometimes see is we end up creating a co-dependency with patients when they have a care coordinator instead of doing the very thing we are trying... We should be teaching them[patients] how to self-manage and everything we do should be preparing them for discharge so they can learn how to do things within the system as part of the skill set that they are leaving with and if we are always doing it for them, or they are always relying on the care coordinator, they are not ever having to do that [care management] on their own.”*

*P* Primary Care Provider, *A* Administrators, *I* Insurers

### Theme 1: A defined scope, rationale, and key partnerships for building comprehensive programs

#### Care coordination inclusion and exclusion criteria

All three stakeholder groups recognized the significance of care coordination and identified the need to have a clear rationale and principles underlining the program. Respondents alluded to the lack of standardized patient inclusion/exclusion criteria which poses a challenge to identifying patients eligible for care coordination programs. Examples of patient characteristics considered most appropriate for care coordination include medical (diabetes, chronic obstructive pulmonary disease (COPD), cardiovascular, kidney diseases), behavioral (non-compliance with care plan, frequent users of urgent care or ER), psychological (depression), and/or social factors (low income, transportation and housing needs, lack of family or caregiver support). There were conflicting perceptions around patient adherence and the question of focusing on ‘rising risk’ and overly complex patients who might not progress. Conceptually, care providers and insurers considered non-adherence as an important criterion for inclusion in care coordination but the same characteristic was also perceived as a potentially undermining factor in some cases. Also, there was some disagreement in expanding care coordination inclusion criteria beyond the COMPASS model, about the most psychiatrically complex patients with some indicating that those patients absolutely should be included while others felt they would be inappropriate for care coordination because of the complexity of their chronic psychiatric needs which may exceed the services care coordination could provide.

Respondents talked about timing and the need to have an entry and exit points for patients. More than half of respondents, regardless of stakeholder group, felt care coordination could be both time limited and ongoing, and dependent on patient needs. Providers tended to lean towards ongoing care coordination for patients. Also enrollment criteria must be flexible in adjusting to patient needs such as end-of-life care, and fluctuating levels of care intensity at different time periods, depending on the state of a patient’s health or their ability to self-manage.

#### Care coordination as a vehicle to bring together medical and community resources for complex patients

These findings focus on a plan for an integrated and comprehensive care coordination program. All stakeholder groups identified that fragmentation of systems and fragmentation of communication among health care providers make coordinating care extremely challenging. Consequently, respondent groups strongly favored approaches that include broad-based multidisciplinary and diversified programs which have both medical and social-service related components. Primary care providers especially expressed that the inability to meet socioeconomic needs of patients is a huge challenge to care coordination, as many eligible patients have extenuating factors inhibiting their participation. Such needs include nutritional, financial, psychosocial, housing, language, and transportation. Considerations of these needs and connecting patients to the available resources are essential to care coordination. Stakeholder engagement was seen to include support and commitment from leadership and clinic “champions” that can assist with program sustainability and expansion.

### Theme 2: Effective information exchange

This theme focuses on the need for all stakeholders, care teams and caregivers involved in patient’s care to have access to relevant and appropriate information through interpersonal-level interactions, technology and health system information.

#### Theme 2.1. Strong interpersonal level-interactions among stakeholders, and with patients

All stakeholder groups acknowledged that communication between and among multidisciplinary care teams both at a clinical level as well as outside clinical care can be challenging lacking centralized care plans. They noted that lack of communication between insurers, social services, community resources, and health care creates duplication of efforts and confusion. Having strong and good working relationships among care teams and co-locating care teams and care coordination teams were identified as some ways to facilitate communication and enhance care coordination.

Insurers in this study acknowledged a lack of awareness of care coordination presence within practices, while administrators and primary care providers commented on a lack of trust among care teams, as issues which impede a successful comprehensive program. Communication across stakeholders was reported as essential to address some of these issues and will enhance professional trust, distribute workloads, and improve patient engagement.

#### Theme 2.2. The need for data and access to an interoperable health information system

Support for new technology and information systems for real-time access to and tracking of patient plans was identified as an important component for an effective care coordination program, particularly among health care administrators. Stakeholders reported on using alternative technology for communication with patients. The perceived technology needs ranged from the ability to video conference with patients to having streamlined information systems and a robust electronic medical record, strong analytic tools, and integrated systems to allow for communication between all providers, the patient, and possibly the health plan.

Stakeholders also discussed the need for technical and information-system support for identifying patients, managing and tracking patients and ensuring appropriate use of resources. A more integrated and encompassing electronic medical record that has real-time ability to show updated medications, see how patients are moving through care, allows for a single plan of care, has multidirectional flow with those inside and outside of the medical setting and possibly allows linking with family members were reported as necessary.

### Theme 3: Trained and available workforce

Effective and well-trained care coordinators emerged from stakeholders as an essential factor that needs to be in place for care coordination implementation. Stakeholders perceived that there is a lack of skilled, specialized and experienced care coordination staff. Required skills for care coordinator positions include both medical and social work aspects along with an ability to build effective relationships with patients. Due to perceived lack of easy availability of skilled staff, hiring and retaining the right care coordinator is important. Competent care coordinators were described as those having specialized skillsets in addition to having the “personality” (being motivated and interested) to be able to manage complex patients. The ability to communicate effectively with providers, patients, families, peers, and community resources was identified as a key trait of an effective care coordinator.

Stakeholders said that care coordinators must be embedded in the medical practice and there should be a clear definition and delineation of roles and scope of practice to prevent duplication of efforts. Setting boundaries for staff and care coordinators were expressed strongly by administrators. One administrator gave an example of registered nurses routinely providing counseling to patients, which was not a part of their defined role.

### Theme 4: Need for a business model and financially justifiable program

Stakeholders strongly expressed that current financial models do not generate adequate direct revenue, which can be a significant impediment to care coordination sustainability. Cost sharing by patients was discussed as an alternative to sustaining care coordination but respondents recognized that such a model can drive patients to opt out of care coordination. While primary care providers appear to advocate for cost sharing, administrators suggested its impracticality. They suggested that for care coordination to be successful there needs to be payer reform, or at least a restructuring of reimbursement. In particular, the perceived cost of the personnel required to provide care coordination was mentioned by a notable portion of respondents as a barrier to implementing an effective and sustainable care coordination program. They noted that it may be expensive to financially support care coordinators and it is *“a very intensive job that requires a lot of time and attention”* which needs to be financed appropriately to attract and retain talented staff.

### Theme 5: Evaluation and improvement of care coordination programs

The capability to evaluate care coordination effectiveness was perceived by all stakeholders as imperative to measure impact on patient health, care experience, and quality of life. All stakeholder groups spoke of lack of an integrated, reliable care management tool capable of tracking patient information and outcomes. Currently there is also a lack of integration with health systems, records and other patient information databases. Care management services that include being able to track and measure patient outcomes, personnel evaluation, service evaluation, as well as a culture of transparency and openness were discussed. In most medical environments, the record keeping system is not designed to monitor population outcomes, fidelity to a treatment model, and cost outcomes. A system that can merge clinical outcomes with utilization outcomes was seen as ideal.

Regular evaluation was reported as essential to streamline processes that are less effective, recognize service gaps that might otherwise go undetected, and to document the impact of care coordination programs. There was mention by all respondent groups that capturing any impact that care coordination had on reducing healthcare utilization costs would be important in supporting continuation of care coordination programs and expanding them. Stakeholders acknowledged that measuring impact can be complex but having systematic way of identifying and tracking patient outcomes and processes via technology can inform policymakers, insurers, and other stakeholders.

### Theme 6: Patient and family engagement

Patient and family involvement in the care coordination were described as an important component for care coordination program. Similar to primary care provider engagement, stakeholders in this study reported that patients’ willingness to participate in care coordination is essential. Many stakeholders described past experiences with patients who were eligible for care coordination but unwilling to participate. They emphasized that the work involved in getting patients and family members engaged “*patients who really need help but are not interested*” can be daunting (see Table theme 6).

Patient characteristics such as minimal motivation and interest, unfamiliarity with existing resources and services, financial need, health state and access limitations were mentioned as factors that could inhibit participation and continued utilization of care coordination. As a component of care coordination, stakeholders identified the need to address patient activation and motivation. They agreed that having a care coordinator to help identify patients’ goals (as opposed to provider goals) and a course of action to achieve them is imperative in obtaining patient engagement.

Stakeholders expressed concern voiced about a potential over-dependency on care coordination as a barrier to self –management. Identifying and engaging family or other caregivers who could work with care coordinators were discussed as a way to enhance patient’s adherence and limit dependency on care coordinators.

## Discussion

This study is unique in soliciting the views of insurers in a time when health practices are implementing diverse models of care coordination [10] with limited financial support. When asked about key elements of care coordination in a setting of limited resources, our participants (primary care providers, administrators, and insurers) had very similar responses.

Areas of general uncertainty were present on the eligibility of those with significant mental health issues, socioeconomic problems, and those patients unable or unwilling to be engaged. There was also uncertainty about how long care coordination should be offered—is it short term or ongoing? There was consensus that care coordination requires effective electronic tools to support both communication and tracking of health information (within the healthcare systems) and utilization information (from insurance systems). To best address the varied needs of patient groups, a care coordinator would need to have training and skills to address both medical and social needs while not creating dependency in their patients. Insurers were no different than primary care providers and administrators in recognizing the importance of outcomes beyond utilization; including health measures, patient care experience and quality of life. All three groups acknowledged the need for a viable financial model and the potential risk of patient having to share the cost of care coordination if required by that patient’s insurance company leading to a lack of participation in care coordination of high cost patients.

These findings reinforce that respondents were aware of the value care coordination can have in reducing fragmentation [2, 20] and improving health outcomes [15, 21] that are broader than cost containment. Reimbursement models that are strictly fee-for service create a barrier to these important goals [9, 10]. This research supports the idea that both practices and insurers see the value of care coordination and largely agree on the need to design a new payment system.

This study involved one state and included only private insurance providers and practices recently involved in implementation of an evidence-based model of care coordination that included both mental health and medical conditions. This may limit generalizability to groups unfamiliar with care coordination and to where patient populations and payment systems differ.

## Conclusions

Previous qualitative research has included input from primary care providers [21–24], other physicians [9], administrators [22–25], and national experts [9], but to our knowledge there are no previous published examples on this topic that compare input from those working in clinical practice with those working within insurance organizations. When faced with a service that has clear evidence of improvement in patient satisfaction and clinical outcomes, but weaker evidence of reducing utilization of high-cost services [26], healthcare systems may speculate on how to best design their program to attract funding from insurance companies. In this work there was considerable consensus between primary care providers, administrators, and insurers on the value of care coordination to patients, the critical components, and on designing models around a broader set of outcomes than cost containment alone. In the US, care delivery models are impacted by the wide variety of insurance models for patients seen by a given practice. Medicare has recognized the value of care coordination in offering new billing codes for care coordination [27]; the time is ripe for commercial insurance in the US to do the same.

## Additional files

Additional file 1: COREQ Checklist 2. (DOCX 20 kb)

Additional file 2: Care Coordination Interview Guide. Questions used in interviews. (DOC 26 kb)

## Abbreviations

CMS: Centers for Medicare and Medicaid Services
COMPASS: Care of Mental, Physical and Substance-use Syndromes
COPD: Chronic obstructive pulmonary disease
ICSI: Institute for Clinical Systems Improvement
MCHA: Minnesota Comprehensive Health Association
QRS: Qualitative Research Services

## Footnotes

1 SNG Research Corporation has been assisting businesses in Minnesota in doing customized market research since 1984. SNG was contracted to conduct interviews with stakeholders only and were by no means involved in the data analysis, interpretation or writing of this manuscript. In addition, none of the authors are a part of the SNG Corporation.
