# Supplementary material for: Sustainable care coordination: a qualitative study of primary care provider, administrator, and insurer perspectives
Source: BMC Health Serv Res. 2019 Feb 1;19:92. doi: 10.1186/s12913-019-3916-5 (PMC6359857; doi:10.1186/s12913-019-3916-5)
Supplement: Supplementary file 1 — COREQ Checklist 2. (DOCX 20 kb) [file 12913_2019_3916_MOESM1_ESM.docx]

| **Domain 1: Research team and reflexivity** |  | Location in Manuscript (section) |
| --- | --- | --- |
| **Personal Characteristics** |  |  |
| 1. 1. Interviewer/facilitator: Which author/s conducted the interview or focus group? | SNG Research group | Methods-data collection |
| 1. 2. Credentials: What were the researcher’s credentials? E.g. PhD, MD | MD, PhD, MPA, MPH | - |
| 1. Occupation: What was their occupation at the time of the study? | Medical practitioners, researchers and analysts | Methods |
| 1. Gender: Was the researcher male or female? | Mix of both genders | - |
| 1. Experience and training: What experience or training did the researcher have? | Researchers have years of experience in population health, epidemiology, qualitative research | Methods |
| **Relationship with participants** |  |  |
| 1. Relationship established: Was a relationship established prior to study commencement? | Yes | - |
| 1. Participant knowledge of the   Interviewer: What did the participants know about the researcher? e.g. personal goals, reasons for doing the  research | During study recruitment and consent the purpose of the study was explained to potential respondent. Respondents were informed of who the investigators are and the objectives of the study, who will be conducting interviews etc. | - |
| 1. Interviewer characteristics: What characteristics were reported about the interviewer/facilitator? e.g. Bias, assumptions, reasons and interests in the research topic. | The interviewer is a contracted service based group. Neither conflicts of interest nor any interviewer-related  biases were identified. | - |
| **DOMAIN 2: Study Design** |  |  |
| **Theoretical framework** |  |  |
| 1. Methodological orientation and theory: What methodological orientation was stated to   underpin the study? e.g. grounded theory, discourse analysis, ethnography, phenomenology, content  analysis | We used an applied qualitative research, and an integrated approach to analysis. Our purpose is neither theory building nor theory confirmation. Moreover, in the broader traditions of qualitative research, it is not essential to have a theory or impose a framework on the data. There is a great deal of work focusing on this aspect. For example, Graebner ME, Martin JA, Roundy PT. Qualitative data: Cooking without a recipe. Strategic Organization. 2012 Aug;10(3):276-84 | Study design and setting.  Analysis |
| **Participant Selection** |  |  |
| 1. Sampling: How were participants selected? e.g. purposive, convenience, consecutive, snowball | A purposive sampling approach using criterion-based and snowballing techniques | Respondent and recruitment |
| 1. Method of approach: How were participants, approached? e.g. face-to-face, telephone, mail, email | Email and follow up phone calls | Respondent and recruitment |
| 1. Sample size: How many participants were in the study? | 42 | Respondent and recruitment |
| 1. Non-participation: How many people refused to participate or dropped out? Reasons?   Setting | 69 PCP did not respond to our emails. 11 administrators did not respond to our emails. There were no respondents from insurer group  who subsequently  refused to  participate, withdrew  consent or dropped  out. | Respondent and recruitment |
| Setting |  |  |
| 1. Setting of data collection:   Where was the data collected? e.g. home, clinic,  workplace | Data were collected over the phone. | Data collection |
| 1. Presence of non-participants:   Was anyone else present besides the participants and researchers? | No | - |
| 1. Description of sample: What are the important characteristics of the   sample? e.g. demographic data, date | We considered different roles of the respondent as adequate to the study and hence our focus. |  |
| **Data Collection** |  |  |
| 1. Interview guide: Were questions, prompts, guides provided by the authors? Was it pilot tested? | Interviews guides were  semi-structured  using probes and follow up for the different respondent groups. | Data collection |
| 1. Repeat interviews: Were repeat interviews carried out? If yes, how many | No | - |
| 1. Audio/visual recording: Did the research use audio or visual recording to collect the data? | Audio recording | Data collection |
| 1. Field notes: Were field notes made during and/or after the interview or focus group? | No additional field notes. |  |
| 1. Duration: What was the duration of the interviews or focus group? | Mean time was 22 minutes | Results |
| 1. Data saturation: Was data saturation discussed? | Yes | Data collection |
| 1. Transcripts returned: Were transcripts returned to participants for comment and/or correction? | No | - |
| **Domain 3: Analysis and findings** |  |  |
| **Data Analysis** |  |  |
| 1. Number of data coders: How many data coders coded the data? | 3 | Analysis |
| 1. Description of the coding tree: Did authors provide a description of the coding tree? | Coding is described in methods section | Analysis |
| 1. Derivation of themes: Were themes identified in advance or derived from the data? | Data were analyzed using an integrated approach. | Analysis |
| 1. Software: What software, if applicable, was used to manage the data? | Nvivo | Analysis |
| 1. Participant checking: Did participants provide feedback on the findings? | No | - |
| **Reporting** |  |  |
| 1. Quotations presented: Were participant quotations presented to illustrate the themes / findings? Was each quotation identified? e.g. participant number | Yes. Direct quotes are presented. | Table |
| 1. Data and findings consistent: Was there consistency between the data presented and the findings? | Yes. Presented as themes. | Results |
| 1. Clarity of major themes: Were major themes clearly presented in the findings? | Yes | - |
| 1. Clarity of minor themes: Is there a description of diverse cases or discussion of minor themes? | No | - |
