# Supplementary material for: Sustainable care coordination: a qualitative study of primary care provider, administrator, and insurer perspectives
Source: BMC Health Serv Res. 2019 Feb 1;19:92. doi: 10.1186/s12913-019-3916-5 (PMC6359857; doi:10.1186/s12913-019-3916-5)
Supplement: Supplementary file 2 — Care Coordination Interview Guide. Questions used in interviews. (DOC 26 kb) [file 12913_2019_3916_MOESM2_ESM.doc]

**Sustainable Care coordination: Primary Care provider, administrator and insurer perspectives**

SET UP ABOUT COMPASS – same for all groups:

*This project is being done towards the end of a three-year research study across the United States on the implementation of a care coordination model for patients with evidence of instability in both medical (cardiovascular disease and/or diabetes) and psychiatric (depression) disorders. As the COMPASS study comes to a close, many of the medical groups involved are debating the future of care coordination in their settings.*

SCREENING QUESTION:

**Script for Administrator:** *Before we begin, your name was provided as someone involved in an administrative role as it pertains to implementing or sustaining care coordination models like COMPASS. Is this accurate?*

**Script for PCP:** *Before we begin, your name was provided as a primary care provider with experience in having patients enrolled in care coordination (COMPASS and others if applicable). Is this accurate?*

**Script for Insurer:** *Before we begin, your name was provided as a ‘go to’ person in your organization for questions about the quality and viability of care coordination programs. Do you feel you have knowledge and experience to answer questions on this topic?*

**Introduction:**

For the purpose of this interview, please imagine that you are asked to provide input to a medical group on how and where to invest their resources for care coordination. In an ideal world we might consider care coordination for all patients. However, that is not feasible. So in the following questions, please keep in mind the real world constraints.

1. First, what types of patients, if any, do you think are not appropriate for care coordination? Why are they not appropriate?
2. Please describe what type of patient you would consider appropriate for a care coordination program.
   1. What medical, behavioral, psychological, social factors (if any) would you consider?
3. For patients you believe would be appropriate for care coordination, what would you envision care coordination to look like?
   1. What types of clinical supports would be available?
   2. Who should be involved in the care coordination program and what should their roles be?
   3. Should care coordination be a time limited or an ongoing option for patients? Why or why not?
4. From your perspective, what are the essential or critical outcomes that you would expect from a care coordination program?
5. What are the essential factors that need to be in place for care coordination to be successfully implemented?
6. What barriers, if any, do you see to implementing an effective and sustainable care coordination program?
